# Virus and viral components transmitted through surgical smoke; a silent danger in operating room: a systematic review

**DOI:** 10.1186/s12893-024-02514-z

**Published:** 2024-08-09

**Authors:** Bahareh Mahdood, Amirmohammad Merajikhah, Mina Mirzaiee, Maryam Bastami, Sara Banoueizadeh

**Affiliations:** 1 Department of Operating Room, Faculty Member of Paramedical School, Jahrom University of Medical Sciences, Jahrom, Iran; 2 Department of Operating Room, Sabzevar University of Medical Sciences, Sabzevar, Iran; 3 Department of Operating Room, School of Paramedical Science, Hamadan University of Medical Sciences, Hamadan, Iran; 4 Department of Operating Room, School of Allied Medical Sciences, Ilam University of Medical Sciences, Ilam, Iran

**Keywords:** Surgical smoke, Virus, Smoke-generating device, Viral components

## Abstract

**Background:**

During surgical procedures, heat-generating devices are widely used producing surgical smoke (SS). Since the SS can transmit infectious viruses, this systematic review was designed to investigate the potential viruses transmitted through SS.

**Methods:**

PubMed, Scopus, Web of Science, ProQuest, and Embase databases, along with Cochran Library, and Google Scholar search engine were searched systematically (by April 21, 2024). No language, place, and time restrictions were considered. All studies evaluating the SS and virus transmission, and whole investigations regarding the viral infections transmitted through SS were totally considered inclusion criteria. Besides, non-original, qualitative, case reports, case series, letters to the editor, editorial, and review studies were excluded from the analysis. This study was conducted in accordance with the PRISMA 2020 statement.

**Results:**

Twenty-six eligible studies were selected and reviewed for data extraction. The results showed that the SS contains virus and associated components. Six types of viruses or viral components were identified in SS including papillomavirus (HPV, BPV), Human Immunodeficiency Virus (HIV), varicella zoster, Hepatitis B (HBV), SARS-CoV-2, and Oral poliovirus (OPV), which are spread to surgical team through smoke-producing devices.

**Conclusions:**

Since the studies confirm the presence of viruses, and viral components in SS, the potential risk to the healthcare workers, especially in operating room (OR), seems possible. Thus, the adoption of protective strategies against SS is critical. Despite the use of personal protective equipment (PPE), these viruses could affect OR personnel in surgical procedures.

## Introduction

Following the progress of surgical procedures, several energy-generated devices are utilized in ORs [1, 2]. Electrocautery is the most common heat-generating device that hires high-frequency electric current to cut or coagulate tissues [3]. Todays, electrocautery, laser, and ultrasonic scalpel are widely recognized as important advances in surgical procedures. It is increasingly used for tissue cutting, hemostasis [4–6], surgical cutting, and tissue separation. Also, laser, ultrasonic scalpel, and electrocautery are used for coagulation of small blood vessels. The main feature of these techniques is the induction of high temperatures causing the burn and rupture of cell membranes and other structures of tissue [7]. SS is a byproduct of bioaerosols produced by energy devices during cutting or coagulation which contains a lot of dangerous components [1, 8]. SS is comprised of 95% of water vapor and 5% of particulate matter active viruses [9] identified as potential hazards for surgical room staff [10, 11]. Inhalation of surgical fumes could be dangerous for the patient and all members of the surgical team, OR nurses, physicians, and surgical technologists [12]. Small inhalable components are discovered in SS. SS also includes several gaseous particulates with the potential of cancer induction. Evidences show that daily inhaled SS particles is equivalent to smoking ten cigarettes [13]. The smoke of the particles is not visible and the smell is unpleasant [12]. Many types of viruses have been detected in SS which are produced in different surgical procedures [13]. Some studies confirmed the transmission of various viruses through SS [14–16]. SS is created with or without during a heating procedure, including bioaerosols with living and non-living cellular materials, which subsequently induce the risk of infection with the virus, and lung irritation, leading to acute and chronic inflammatory changes. [17, 18].

Garden et al. showed that HPV can be identified from SS in dioxide lasers during gynecological procedures [19]. Also, Zhou et al. (2019) approved the presence of DNA of human papilloma available in SS which can be transferred from patients to the members of surgical team [20]. Also, Parker et al. reported that a gynecological surgeon was infected with HPV through SS. [21] Surgical personnel are exposed to these chemicals for an average of 7 h per day, 5 days per week, and throughout the period of several years [22] and smoke production during surgical procedures is unavoidable [23]. Although SS can induce unavoidable side effects for the surgical team as well as the patients, these side effects are not fully discovered. One of these complications is the transmission of viruses through SS. Since the surgical team members may not be aware of this risk, they are more vulnerable. In the meantime, it should be noted that the utilization of PPE supply is not sufficient safety against SS [17]. Also, the personnel widely use surgical masks, which are ineffective in protecting against the effects of SS [24]. As a result, it is necessary to be aware of viruses transmitted through SS and to adopt methods preventing the transmission of infection in this regard.

## Materials and methods

### Data collection

The PRISMA flowchart and the associated checklist were utilized to assess and identify the types of viruses transmitted through SS [25].

### Search strategy

The related studies were searched using PubMed, Scopus, Web of Science, ProQuest, Embase, Cochran Library and Google Scholar search engine. The studies were compiled by April 21, 2024, with no language, place and time limitation. The search was applied using the keywords of (surgical smoke) OR (surgical plume) OR (aerosol) AND (electrocautery) OR (diathermy) OR (laser) OR (scalpel harmonic) AND (viral infection) OR (Virus) AND (Complications) AND (DNA) OR (DNA transmission) OR (RNA) OR (RNA transmission).

To conduct the search protocol, two authors initially reviewed the sources of qualified article reports and subsequently evaluated the Abstracts and Titles of the identified articles. 1126 duplicate articles were found and merged. Unrelated and non-original articles were excluded from the study. The related data, device, type of article, surgical procedure, and viral type were identified using separate authors.

### Inclusion and exclusion criteria

Inclusion criteria were the studies regarding the expression of SS and virus transmission and the investigations about viral infections transmitted through SS. Non-original, qualitative, case report, case series, letters to the editor, editorial and review studies were excluded from the analysis. Also, the studies with no report of the presence of any viral components in SS were excluded.

### Data extraction

Article selection process was conducted by two researchers (BM, MM), independently. In case of disagreement, the third author (AM) was responsible for final agreement. For the data extraction process, an electronic datasheet including the year of publication, first author`s name, study design, device, and viral component was used.

### Quality assessment

Based on the guidelines provided by the Newcastle and Ottawa declarations, the methodological quality of the articles was evaluated [26]. In this guideline, a confirmed framework for quality assessment of the articles is used. In this guideline, criteria were considered for the selection of study subjects, comparison, exposure, and their results, and a maximum of 9 stars were assigned to each study. Studies > 7 stars were classified as high quality and studies < 6 stars had low quality. In order to avoid probable bias, the results of the study were independently checked by two researchers. In case of disagreement, the authors resolved it with negotiation. This approach provided a complete assessment of possible bias in the results.

## Results

**Fig. 1:**
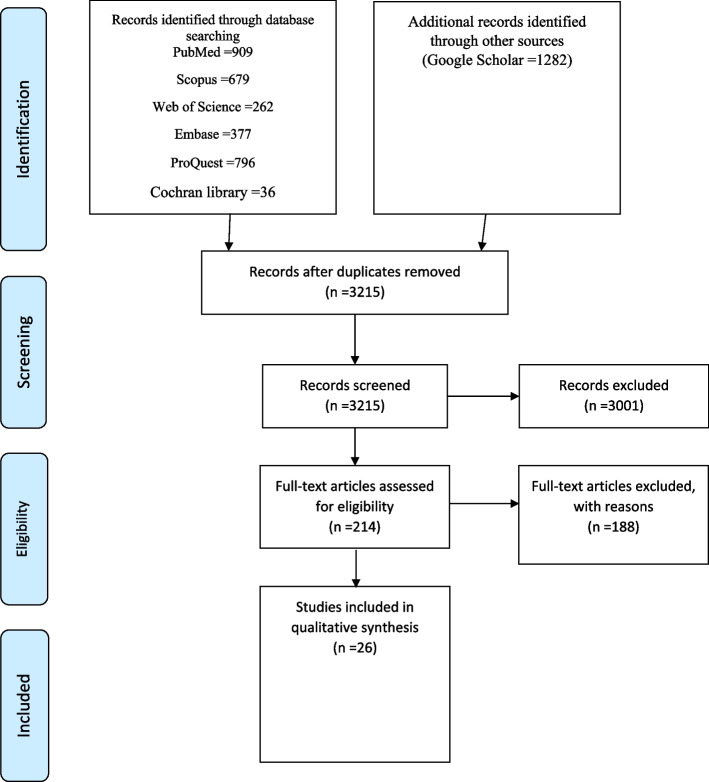
Flow diagram of the study selection for the review process

SS is frequently inhaled by surgical team members in OR. The particles in this smoke can be dangerous for healthcare workers and may contain pathogenic microorganisms. Thus, the present study examined the viruses transmitted through SS. In the initial search, 4341 articles were found in PubMed, Scopus, Web of Science, ProQuest, Embase, Cochran Library, and Google Scholar. These studies were compiled by April 21, 2024. Following the paper checking by Endnote software (v.8x), 1126 duplicate articles were detected. By examination of the Titles and Abstracts, 3001 records were removed and 214 articles remained for full-text study. In the next step, 188 full-text articles were removed and 26 full-text articles were reviewed, based on the inclusion/exclusion criteria. The remaining 26 articles included 5 clinical trials, 4 prospective studies, 15 in vitro-in vivo-ex vivo, and 2 descriptive studies (Fig. 1 and Table 1).

**Table 1:** viral type in surgical smoke

NO	Author	Devise	Type of article	Surgical procedure	viral type
1	Wesley Pereira Andrade, 2020 [27]	Monopolar electrosurgical	RCT	Mastectomy (Cancer)	COVID‐19
2	Bogani, G, 2021 [28]	Monopolar electrosurgical	A prospective pilot study	All laparoscopic surgery for gynecologic malignancies (Cancer)	SARS-CoV-2
3	Marc Garbey, 2020 [29]	Monopolar and bipolare electrosurgical	In vitro	In vitro	SARS-CoV-2
4	Xiaoli Hu, 2021 [18]	loop electrosurgical excision procedure(LEEP)	RCT	Gynecological surgery	HPV
5	Gregory K. Johnson, 1991 [30]	Electrocautery, Spinning router tip, Stryker oscillating bone	In vitro	In vitro	HIV-1
6	Han Deok Kwak, 2016 [31]	Electrosurgical devices	RCT	Laparoscopic or robotic abdominal surgeries	HBV
7	William S. Sawchuk, 1989 [32]	Carbon dioxide laser Electrocoagulation	RCT	Biopsy resection of foot	HPV, Bovine papillomavirus
8	Wisniewski, P. M, 1990 [33]	CO2 laser	In vitro- in vivo	Gynecological surgery	HPV
9	Linzhi Yan, 2022 [34]	Ultrasonic scalpel	In vitro- in vivo	Cervical cancer xenograft tumors Laparoscopic hysterectomy	HPV
10	Takuya Yokoe, 2021 [14]	Electric scalpel Ultrasonic scalpel	In vitro	In vitro	SARS-CoV-2
11	Taravella, Michael J, (1999) [35]	Excimer laser	In vitro	In vitro	OPV
12	Kay Neumann, (2018) [16]	loop electrosurgical excision procedure (LEEP)	The prospective pilot study	Gynecosurgery and Obstetrics	HPV
13	Zhou Q, (2019) [20]	loop electrosurgical excision procedure (LEEP)	RCT	Gynecosurgery	HPV
14	Garden, Jerome M, (2002) [36]	Carbon dioxide laser	In vitro	Design Bovine papillomavirus–induced cutaneous fibropapillomas were exposed to the carbon dioxide laser	papillomavirus DNA in all tested laser settings viral DNA was most likely encapsulated
15	Bogani, G. (2021) [28]	Not specified	A prospective pilot study	laparoscopic procedures	SARS-CoV-2
16	Cizmic, Amila (2023) [37]	Not specified	prospective, single-center clinical trial	Minimally and open surgery	Any viruses have not identified
17	Garden, Jerome M (1988) [19]	carbon dioxide laser	In vitro	• an in vitro cutaneous bovine fibropapilloma • an in vivo human verruca model	HPV DNA
18	Hirota, M. (2022) [38]	• Ultrasonically activated scalpelel Ectrocautery	ex-vivo clinical specimen	• ex-vivo model: Tumor mass of a hepatocellular carcinoma line was prepared in mouse • clinical specimen: Detection of HBV-DNA and HBsAg was conducted following the collection of surgical plume generated from clinically obtained liver specimens	• HBs gene identified • HBV DNA
19	Hu, Xiaoli (2021) [39]	loop electrosurgical excision procedure (LEEP)	In vivo and cross sectional	In vivo	HPV
20	Hughes, Philip SH (1998) [40]	erbium:YAG laser	In vivo	Removal of human papilloma warts	• HPV absence in laser plume • HPV DNA in laser plume
21	Yokoe, T (2021) [14]	blade of the Harmonic ultrasonic surgical device bipolar electrosurgical scalpel	In vivo	HeLa-ACE2-TMPRSS2 cells infected with human coronavirus	Human coronavirus RNA
22	Yan, Linzhi (2022) [34]	ultrasonic scalpel	In vivo	cutting and coagulation of cervical cancer xenograft tumors	• HPV DNA
23	Taravella, Michael J (1997) [41]	Excimer laser ablation	In vivo	Ablation of Human embryonic lung fibroblasts infected with attenuated varicella-zoster virus	• varicella-zoster DNA
24	Llueca, Antoni (2021) [42]	Not specified	cross-sectional	Laparoscopy (digestive surgery, gynaecologic surgery, urologic surgery.)	• SARS-CoV-2
25	Kwak, H. D (2016) [31]	Electrosurgery	Descriptive-analytical	laparoscopic or robotic abdominal surgeries	• HBV
26	Johnson, G. K. (1991) [30]	• Electrocautery • High speed bone cutting router • Stryker oscillating bone	In vivo	HIV-infected culture medium	• SS generated by electrosurgery: No HIV-1 • SS generated by surgical power devices: HIV-1 identified

The results showed that the SS contains the virus and associated components. Finally, 26 eligible studies were reviewed. Six types of viruses and the associated components were identified including Human papillomavirus (HPV), Human Immunodeficiency Virus (HIV), Hepatitis B (HBV), SARS-CoV-2, Oral poliovirus (OPV), and varicella-zoster which are spread through smoke-producing devices (Table 1).

Eleven studies approved the presence of papillomavirus in SS. Also, 7 investigations of SARS-CoV-2, 3 studies of HBV, 2 studies of HIV, and 1 study for OPV and varicella-zoster were identified. Also, 1 study reported no identified virus. The use of heat-generating devices in surgeries causes SS, and according to the analyses, some types of viruses were found in SS.

## Discussion

The results of this systematic review verified the viruses and viral components transmitted through SS. Since electrosurgical devices are commonly used in operations globally, the SS is considered a health risk. Smoke-generating devices include Electrocautery and Electrosurgery, Ultrasonic Ablation, High-speed Burs, Drills, Saws, Lasers, and Harmonic scalpels. Among these devices, electrocautery is the most common tool used in OR, which is considered an essential tool in modern surgeries in all ORs [24, 43, 44]. Approximately, 500,000 healthcare providers (such as surgeons, nurses, anesthesiologists, and technicians) are exposed to SS in ORs per year [45]. Several studies reported the presence of viral genomes in SS [30, 31, 46]. Also, some articles showed the existence of virus DNA in SS [19, 32].

Our study revealed 6 types of viruses and viral components in SS.

### Papillomavirus

HPV and bovine papillomavirus (BPV) can be spread in the air through SS leading to human diseases. However, according to several papers, HPV is not found in SS and the SS infection threat is low levels or impossible [47, 48]. Also, recent studies approved the presence of HPV in SS [20, 39]. On the other hand, Xiaoli Hu et al. indicated that gynecologists using electrosurgery such as LEEP are at risk of HPV infection [39]. According to a case report, a 66-year-old gynecologist with the experience in approximately 500 electrosurgical procedures was reported for HPV-related cervical dysplasia and vulvar lesions over a 40-year period. [21].

Surgical face masks, particularly the N95, reduce the hazard of HPV transmission. On the other hand, gynecologists and surgical technologists are at risk of this disease. Stefano Palma et al.'s study also confirmed the spread of this virus following the application of LEEP and CO2 laser. Also, this study reported the cases of upper airway neoplasms due to HPV transmission through SS [49]. DNA of papillomavirus has been identified from laser plumes from papilloma lesions, and nasal papilloma is detected to develop in the noses of OR staff and physicians exposed to laser smoke [19].

Since this virus is very contagious and prevalent leading to benign dermatological and anogenital warts, oropharyngeal and laryngobronchial lesions, cervical cancer, mouth, and laryngeal cancer, depending on the genotype of the virus. Although the transmission of this virus is applied mainly through intercourse, recent studies confirmed the SS as a transmission route [39, 50].

William S. Sawchuk et al. reported the spread of papillomavirus DNA from carbon dioxide laser smoke and tissue coagulation in leg biopsy resection surgery [32]. Because this virus has different genotypes, the dangerous genotypes in SS can increase the risk of developing malignant cancers in healthcare workers. Transmission of this virus with SS is more common in gynecological procedures than in other surgeries.

The utilization of electrocautery and laser by gynecologists is a prominent step in cervical and ovarian cancer surgeries; especially, the LEEP which is used for treatment of precancerous lesions due to the HPV infection causing a critical issue due to the SS produced by these devices. This smoke has many potential risks to gynecologists. Linzhi Yan's study showed that the SS produced during cervical cancer surgery contains HPV with cytotoxicity and infectivity in laboratory conditions [34]. Another case was a 64-year-old gynecologist who was infected by HPV. He performed 250 electrosurgical surgeries for HPV-associated cervical dysplasia and vulvar lesions over a 27-year [21].

Based on the transmission of this type of virus through SS, it is necessary to prevent smoke inhalation and contracting this disease for all healthcare workers exposed to SS. The use of masks, especially N95 and Gardasil vaccination for prevention of this disease seems essential for surgical staff.

### SARS-CoV-2 virus

In early December 2019, a case of pneumonia with unknown origin was identified in Wuhan, China [51, 52]. The causative pathogen was a novel beta-coronavirus with capsular RNA, named Coronavirus 2, which is phylogenetically similar to SARS-CoV [52, 53]. SARS-CoV-2 is highly contagious which affects the respiratory system, causing fever, sore throat, cough, chest and muscle pain, Dyspnea, anosmia, headache, confusion, and ageusia [54–56]. These complications cause life-threatening respiratory failure, as well as affect the heart, kidneys, liver, and nervous system. In addition to the involvement of respiratory system, this virus affects the gastrointestinal system from the mouth to the rectum, blood, saliva, urine, and probably the liver in infected individuals [57–60]. Although this virus spreads through the aerosols in air, our findings showed that it is also transmitted by SS.

Bogani et al. confirmed that this virus could be transmitted through SS and aerosolized fluid from the abdominal cavity through the SS from monopolar electrosurgical units in women's laparoscopic procedures [28]. On the other hand, evidence showed that SS could transfer this virus [29, 50].

In his study, Takuya Yokoe collected SS with a vacuum pump and analyzed it for the presence of SARS-CoV-2 virus RNA. The results of this study showed that human coronavirus RNA is present in SS generated by cutting infectious tissue using an ultrasonic scalpel [14]. Also, Andrade et al. approved the risk of COVID-19 infection for surgical teams in the OR due to long-term exposure to SS [27].

Weissleder et al. showed that sputum and feces from patients infected with SARS-CoV-2 individuals include viral RNA [61]. Thus, incisions made on the intestinal and upper respiratory tract can generate SS containing viral components. Evidence also showed that the risk of active virus transmission through SS is higher in laparoscopic surgeries [62]. Coccolini et al. stated that peritoneal fluid can contain SARS-CoV-2 [63]. Other studies confirmed the presence of this virus in SS aerosols from laparoscopic surgeries [28, 42]. Following the presentation of COVID-19 vaccines, this disease was controlled, however, it is observed in different parts of the world. Thus, more preventive strategies should be adopted in ORs despite vaccination [64].

### HBV

HBV infection is an international health threat, and 2.57 billion people worldwide are affected by HBV. The estimated annual mortality of hepatitis B is more than 780,000 [65–68]. Hepatitis B virus is transmitted through direct contact with infected blood and aerosol form. Our study confirms the existence of the hepatitis B virus in SS.

Han Deok Kwak et al. identified the HBV in SS from electrosurgery in robotic or laparoscopic colorectal resection, laparoscopic gastrectomy, and laparoscopic hepatic wedge resections [31]. This dangerous virus leads to a comprehensive range of hepatic pathologies from acute diseases (including severe liver failure) to chronic hepatitis, cirrhosis, and hepatocellular carcinoma. Although vaccination of healthcare workers with hepatitis B vaccine can effectively prevent this disease, the reduction of antibody titer below the required level of immunogenicity can threaten them. Thus, it is recommended to periodically check the antibody titer in all at risk personnel.

Vourtzoumis et al. reported that HBV exists in SS during laparoscopic procedures in patients [57]. However, these types of studies reporting the presence of HBV are rare. Acute HBV infection can be asymptomatic or with symptomatic acute hepatitis [69, 70]. Thus, some strategies must be adopted to reduce the risk of the virus in health workers.

### HIV

Our study confirms the existence of HIV in SS. Human immunodeficiency virus, the cause of AIDS (Acquired immunodeficiency syndrome), is responsible for the most common epidemic in humans [71, 72]. HIV attacks the immunological system and suppresses the potential activity. This virus is transmitted through intercourse, exposure to infected blood or tissue, and from mother to fetus during pregnancy, childbirth, or breastfeeding. However, the transmission of this virus through SS is discussed.

Although there are a few studies regarding the dangerous condition, the evidence show that HIV is present in the SS from electrocautery causing associated infection [30]. Also, in a laboratory study, Baggish et al. showed that HIV proviral DNA was found in SS produced by CO2 laser [73]. Although epidemiological data regarding the prevalence of HIV infection in different populations show that transmission through aerosols is not common [74–76], the risk of transmission through SS remains a threat to the health of medical personnel. The incubation period of this disease is long-term which can increase the risk of transmission of this type of virus. Because the patient represents no signs during the incubation period, the patient is unaware of the existence of the disease.

HIV virus diagnostic test is not performed prior to all surgical procedures. Thus, the healthcare workers must be aware of this dangerous condition. Other evidence also found the existence of HIV DNA and RNA in SS. Since this virus leads to immunodeficiency as a life-threatening agent, the recognition of the routes of virus transmission is crucial.

### OPV

Poliomyelitis is caused by the poliovirus. Polio is a gastrointestinal disease, mostly with an oral-fecal transmission route. Besides, pharyngeal droplet secretion is also considered another route of virus transmission. Polio virus enters the body through the mouth, then passes through the digestive system tract, and is finally eliminated by feces and oral secretions in several weeks. Viremia occurs following initial multiplication. In the absence of proper immunity and neutralization, the virus may reach the secondary organs, including the central nervous system. In these organs, the multiplication can lead to the destruction of motor neurons and paralysis [77].

Taravella et al. observed the existence of poliovirus in SS created by excimer laser tissue ablation. Oral poliovirus as an RNA-based virus has no lipid envelope. This characteristic in comparison with other viruses (such as herpes) makes it safe from the heat generated by the excimer laser [35]. Since the OPV is mostly rare due to the existence of a vaccine and the awareness of the associated problems, the potential risks are prevented.

### Varicella zoster

Varicella zoster virus (VZV) causes primary infection (varicella or chicken pox) followed by delayed onset of sensory ganglia. The virus can reactivate and cause herpes zoster (HZ, shingles) leading to considerable complications such as death, in rare cases [78]. Following the ablation of human fetal lung fibroblasts infected with attenuated varicella-zoster virus by an excimer laser, Taravella et al. concluded that varicella-zoster DNA is found in laser smoke [41]. Since there are a few studies investigating the presence of this virus or its genetic material in SS, more studies are needed to confirm this issue.

Since the identification of viral DNA in SS is a challenging process, further researches regarding the number of viruses present and their transmissibility are needed.

In the present study, all aspects of transmission of viruses, DNA, and its viral contents were systematically exhibited. Potentially, the risk of transmission seems probable, but there are a few diagnosed cases of HPV infection. This systematic review showed that only HPV infections among healthcare workers are recorded so far; however, the risk of contraction of other viruses cannot be ignored. Many in-vitro/vivo studies identified the associated risk. According to the fact that there is a strict need for more clinical research on other viruses, preventive measures should be applied in ORs.

### How to protect yourself against SS?

Safety is an important content in health procedures [79]. Although general room ventilation (GRV) with positive pressure can reduce the concentration of SS, it is not enough to absorb the pollutants produced by SS. In order to protect the OR staff, surgeons, and other treatment staff from possible dangers of SS, the local exhaust ventilation (LEV) can be used in addition to the GRV in the OR. Two main methods of LEV which can reduce the smoke for surgical team members are portable SS extraction devices and OR suction systems. The SS vacuum should contain the speed of 100–150 feet/minute and the filter should be HEPA (high-efficiency particulate air) or more powerful. These filters must be changed regularly and destroyed along with infectious waste [24]. Surgical room suction systems exhaust smoke at a slower rate. However the use of a smoke-evacuator pencil cautery or an attentive assistant with handheld suction can reduce SS to a greater extent [80].

In addition, the use of highly protective masks (such as N95, N99, N100, P95, P99, P100, R95, R99, and R100) is recommended [81, 82]. Also, one of the most important solutions to control SS is the continuous training of all members of the surgical team regarding the risks and ways to reduce and eliminate SS [83]. Safety and quality are considerable matters while providing healthcare services.

## Conclusion

Utilization of SS-generating devices in surgical procedures is unavoidable. This smoke can threaten the surgical and anesthesia team and even the patients. OR staff are always exposed to SS. This is a chemical and biological hazard. Our research showed that this smoke can contain viruses and non-disease viral components. Some of these viruses can even be considered a threat to a person's life. Educational strategies are crucial to prevent the transmission of infection to healthcare workers through SS. The application of suitable and sufficient PPE can effectively avoid transmission of the virus through SS.

## Data Availability

This published article includes all data generated or analyzed during the study.
